# Genotype Announcement to Japanese Smokers Who Attended a Health Checkup Examination

**DOI:** 10.2188/jea.16.45

**Published:** 2005-12-20

**Authors:** Nobuyuki Hamajima, Koji Suzuki, Yoshinori Ito, Takaaki Kondo

**Affiliations:** 1 Department of Preventive Medicine / Biostatistics and Medical Decision Making, Nagoya University Graduate School of Medicine.; 2 Department of Public Health, Fujita Health University School of Medicine.; 3 Department of Medical Technology, Nagoya University School of Health Sciences.

**Keywords:** Smoking Cessation, Genotype, Disclosure, *GSTM1*, *GSTT1*, *NQO1*

## Abstract

**BACKGROUND:**

Genotype announcement may be one of the effective methods to induce smoking cessation, but the studies are limited throughout the world.

**METHODS:**

Subjects were smokers who attended a health checkup examination provided by a local government in Hokkaido, Japan, 2003. Those who agreed to know their genotypes were informed of the genotypes of *glutathione S-transferease (GST) M1* present/null, *GSTT1* present/null, and *NAD(P)H:quinone oxidoreductase 1 (NQO1)* C609T (Pro187Ser).

**RESULTS:**

Out of 143 smokers (92 males and 51 females), 101 individuals participated in the present study. A postal questionnaire one year after the genotype announcement found that 8 persons (6 males and 2 females) of 41 respondents had quitted smoking. Two of 8 quitters stated that they had quitted smoking due to the announcement. There were none who regretted the genotype tests.

**CONCLUTION:**

Although the cessation rate, 7.9% (8/101) at least, was not marked, no harmful effects were observed among the respondents.

Genotype announcement for smokers is a new approach to induce smoking cessation. To date only two controlled studies in the United States^1^^,^^2^ and one case series study at a Japanese worksite^3^ have been reported in the world. The announced genotypes were *CYP2D6*,^1^ *GSTM1*,^2^ and *L-myc*,^3^ of which functional polymorphisms were reportedly associated with the susceptibility to carcinogens in tobacco smoke.

In the present study, the effects of genotype announcement were examined for smokers who attended a health checkup examination run by a rural local government in Hokkaido, Japan. The announced genotypes were of three polymorphisms, glutathione *S-transferease (GST) M1* present/null, *GSTT1* present/null, and *NAD(P)H:quinone oxidoreductase 1 (NQO1)* C609T. These genes code carcinogen detoxifying enzymes.

## METHODS

Study subjects were sampled from annual checkup examinees run by a local government in Hokkaido on the consecutive three days of August 2003, whose study framework was described in the previous paper.^4^ Informed consent of genotype announcement was obtained individually from each smoker, using a booklet on genotype and susceptibility to tobacco carcinogen. For the smokers who agreed the donation of 0.5ml residual blood, genotypes with an explanation note were mailed 3 months later after the enrollment. Current smoking habits were asked one-year after the announcement by mail. Repeated mailing to the unrespondents was not conducted to giving the potentially unpleasant and troublesome pressure to the participants. *GSTM1* present/null, *GSTT1* present/null, and *NQO1* C609T were genotyped by a triplex PCR with confronting two-pair primers.^5^ The genotype frequency of *NQO1* C609T was in Hardy-Weinberg equilibrium for 131 blood available smokers (CC for 44, CT for 69, and TT for 18, p=0.269). *GSTM1 null*, *GSTT1 null*, and *NQO1 609TT* are genotypes without enzyme activity. Statistical analysis was conducted with STATA^®^ version 7 (STATA Corporation, College Station, TX).

This study was approved by the Ethic Committee of Nagoya University Graduate School of Medicine. (approval number: 48)

## RESULTS

Table 1 shows the age and sex distributions of examinees, smokers, participants, respondents, and quitters. Out of 864 examinees (309 males aged 39 to 88 years, and 555 females aged 39 to 86 years), 143 smokers (92 males and 51 females) were identified from lifestyle questionnaire. Among them, 101 smokers (69 males and 32 females) agreed the genotype announcement. One sample was not available because of the lack of residual blood. The results of genotype testing and a questionnaire were mailed to all participants three months and 15 months after the enrollment, respectively.

**Table 1.** Age and sex distributions of examinees (E), smokers (S), participants (P), respondents (R), and quitters (Q) who attended a health checkup examination in Hokkaido Japan, 2003.

Age (year)	Males					Females				
	
	E	S	P	R	Q	E	S	P	R	Q
39-49	39	20	17	6	0	80	15	13	3	0
50-59	66	26	19	7	0	177	22	15	4	2
60-69	109	28	20	13	3	193	8	4	0	0
70-88	95	18	13	8	3	105	6	0	0	0

Total	309	92	69	34	6	555	51	32	7	2

Of the 101 smokers, 41 (40.6% of 101) responded to the questionnaire. Eight (7.9% of 101 smokers, 95% confidence interval: 3.5-15.0) stated to have quitted smoking; 2 females due to the results of genotype tests, 1 male due to sickness, 4 males due to maintaining health, and 1 male due to no reason. The number of genotypes with no enzyme activity were significantly related to smoking cessation (Fisher’s exact test, p=0.05) between those harboring 0 or 1 genotypes with no enzyme activity (4.3%, 3/70) and those harboring 2 or 3 genotypes with no enzyme activity (16.7%, 5/30). There were no participants who regretted the participation, as shown in Table 2.

**Table 2.** Quitters among participants or respondents.

	Males	Females	Total
Total	6/69 ( 8.7)	2/32 ( 6.3)	8/101 ( 7.9)

Genotypes with no enzyme activity* (One participant’s DNA was not available)			
0	1/16 ( 6.3)	0/ 6 ( 0.0)	1/22 ( 4.5)
1	2/33 ( 6.1)	0/15 ( 0.0)	2/48 ( 4.2)
2	3/17 (17.6)	2/10 (20.0)	5/27 (18.5)
3	0/ 2 ( 0.0)	0/ 1 ( 0.0)	0/ 3 ( 0.0)

How do you feel the genotype tests ? (34 male respondents and 7 female respondents)			
Glad to have participated	4/19 (21.1)	2/ 5 (40.0)	6/24 (25.0)
Should not have participated	0/ 0	0/ 0	0/ 0
Neither	0/ 7 ( 0.0)	0/ 1 ( 0.0)	0/ 8 ( 0.0)
Seems not to have participated	2/ 6 (33.3)	0/ 1 ( 0.0)	2/ 7 (28.6)
Unspecified	0/ 2 ( 0.0)	0/ 0	0/ 2 ( 0.0)

Percentages in parentheses.

*: *GSTM1* null, *GSTT1* null, and *NQO1* 609TT.

## DISCUSSION

Although the response rate was low, this study found that the cessation rate was 7.9% at least. Because this study had no controls, the precise excess cessation due to the genotype announcement was not evaluable. However, two participants stating the cessation due to genotype tests and some of four participants stating the cessation due to maintaining health might be fully or partly induced to quit smoking through the genotype announcement.

Some researchers warn that genotype announcement may be potentially harmful even in the case of polymorphism genotypes. However, there are no reports indicating that the announcement of polymorphism genotypes made serious problems among the announced persons. It is quite different from the announcement of hereditary disease genotypes.

In conclusion, the present study found that about 8% of smokers quitted smoking one year after their genotypes were announced with mail. The effects of genotype announcement on smoking behavior under a face-to-face approach remain to be studied.

## References

[1] Audrain J, Boyd NR, Roth J, Main D, Caporaso NF, Lerman C Genetic susceptibility testing in smoking-cessation treatment: one-year outcomes of a randomized trial. Addict Behav 1997; 22: 741-51. 9426791 10.1016/s0306-4603(97)00060-9

[2] McBride CM, Bepler G, Lipkus IM, Lyna P, Samsa G, Albright J, Incorporating genetic susceptibility feedback into a smoking cessation program for African-American smokers with low income. Cancer Epidemiol Biomarkers Prev 2002; 11: 521-8. 12050092

[3] Hamajima N, Atsuta Y, Goto Y, Ito H A pilot study on genotype announcement to induce smoking cessation by Japanese smokers. Asian Pac J Cancer Prev 2004; 5: 409-13. 15546247

[4] Ito Y, Kurata M, Hioki R, Suzuki K, Ochiai J, Aoki K Cancer mortality and serum levels of carotenoids, retinol, and tocophenol: a population-based follow-up study of inhabitants of a rural area of Japan. Asian Pac J Cancer Prev 2005; 6: 10-5. 15780024

[5] Kawase H, Hamajima N, Tamakoshi A, Wakai K, Saito T, Tajima K Triplex polymerase chain reaction with confronting two-pair primers (PCR-CTPP) for NQO1 C609T, GSTM1, and GSTT1 polymorphisms: a convenient genotyping method. Asian Pac J Cancer Prev 2003; 4: 67-70. 12718704

